# Portfolio Optimization with a Mean–Absolute Deviation–Entropy Multi-Objective Model

**DOI:** 10.3390/e23101266

**Published:** 2021-09-28

**Authors:** Weng Siew Lam, Weng Hoe Lam, Saiful Hafizah Jaaman

**Affiliations:** 1 Department of Physical and Mathematical Science, Faculty of Science, Kampar Campus, Universiti Tunku Abdul Rahman, Jalan Universiti, Bandar Barat, Kampar 31900, Perak, Malaysia; whlam@utar.edu.my; 2 Department of Mathematical Sciences, Faculty of Science and Technology, Universiti Kebangsaan Malaysia UKM, Bangi 43600, Selangor, Malaysia

**Keywords:** entropy, goal programming, optimal portfolio, risk, return

## Abstract

Investors wish to obtain the best trade-off between the return and risk. In portfolio optimization, the mean-absolute deviation model has been used to achieve the target rate of return and minimize the risk. However, the maximization of entropy is not considered in the mean-absolute deviation model according to past studies. In fact, higher entropy values give higher portfolio diversifications, which can reduce portfolio risk. Therefore, this paper aims to propose a multi-objective optimization model, namely a mean-absolute deviation-entropy model for portfolio optimization by incorporating the maximization of entropy. In addition, the proposed model incorporates the optimal value of each objective function using a goal-programming approach. The objective functions of the proposed model are to maximize the mean return, minimize the absolute deviation and maximize the entropy of the portfolio. The proposed model is illustrated using returns of stocks of the Dow Jones Industrial Average that are listed in the New York Stock Exchange. This study will be of significant impact to investors because the results show that the proposed model outperforms the mean-absolute deviation model and the naive diversification strategy by giving higher a performance ratio. Furthermore, the proposed model generates higher portfolio mean returns than the MAD model and the naive diversification strategy. Investors will be able to generate a well-diversified portfolio in order to minimize unsystematic risk with the proposed model.

## 1. Introduction

Complex economic relations and models due to unprecedented events in the ever-evolving financial markets make estimating stock market returns and volatility become complex nonlinear relations, in need of advancements in mathematical models to adapt to changes in the financial markets and mental resources of the investors. The COVID-19 pandemic has led investors to demand portfolios that are resilient towards the disease.

Investors aim to minimize the risk as well as maximize the return in their portfolio investments. In past studies, portfolio management has been analyzed and solved with optimization models [1,2,3]. Markowitz [4] was the pioneer in portfolio optimization by introducing the mean-variance (MV) model to minimize the risk and achieve the investor’s mean return. In MV model, the risk and mean return of investors are measured with portfolio variance and portfolio mean return, respectively. The MV model has been studied in portfolio management according to past research [5,6,7,8,9].

Konno and Yamazaki [10] have introduced the mean-absolute deviation (MAD) model by using absolute deviation in replacement of variance as the portfolio risk measure, because the MV model assumes that the assets’ returns are normally distributed. Konno and Yamazaki [10] have studied the MAD model for the Tokyo stock market. The MAD model has been studied in portfolio optimization [11,12,13,14]. Zenios and Kang [11] have used mortgage-backed securities for portfolio optimization with the MAD model. Zenios [12] has investigated the MAD model for fixed-income securities. Konno [13] has studied the MAD model for small-scale funds. The MAD model has been studied for stock–bond portfolio optimization [14]. Erdas [15] has investigated the MAD model for the Borsa Istanbul 30 Index. Ghahtarani and Najafi [16] have studied the MAD model in portfolio optimization for investments. Kasenbacher et al. [17] have generated the optimal portfolio of stocks with the MAD model. However, the maximization of entropy is not considered in the MAD model of Konno and Yamazaki [10].

Entropy is an important element in portfolio selection [18]. Shannon [19] has introduced entropy as the uncertainty measure. An entropy-based portfolio is constructed based on Shannon entropy. Entropy is a wide measure of diversity [20,21]. According to the principle of maximum entropy, a higher value of entropy for the portfolio will give a higher portfolio diversification, which can reduce the unsystematic risk of the portfolio. Bera and Park [20] constructed a well-diversified portfolio based on the entropy measure for portfolio optimization. Jana et al. [21] incorporated entropy as an objective function in their optimization model, to increase the diversification degree of an optimal portfolio. According to Murialdo et al. [22], the entropy measure generates a high level of diversity and stability in portfolio selection.

Zhou et al. [18] conducted a review on the principles of entropy and their applications in finance, such as portfolio selection. Lu et al. [23] presented an optimization model based on the principle of entropy in portfolio investment. Li and Zhang [24] studied a mean-variance-entropy model by considering the level of diversification in the optimal portfolio. The mean return is measured by expected value, whereas the risk is measured by variance. Entropy is used to measure the level of diversification in the optimal portfolio. The results showed that the level of diversification had an effect on the optimal portfolio investment. The principle of maximum Shannon entropy has been incorporated into the portfolio-optimization model to improve the level of optimal diversification. Therefore, Shannon entropy is used to measure the level of diversification in portfolio selection. Olbryś and Ostrowski [25] introduced a market-depth indicator to investigate the market depth based on Shannon entropy. Other than achieving optimal diversification using a portfolio-optimization model, naive diversification is another investment strategy which aims to reduce the risk measure by forming an equally weighted portfolio, or naive 1/*N* portfolio, without using a mathematical model [26].

The maximization of entropy is not considered in the MAD model according to past studies. Therefore, this paper aims to propose a multi-objective optimization model, namely a mean-absolute deviation-entropy model, by incorporating the maximization of entropy to generate a well-diversified portfolio, to minimize unsystematic risk in portfolio optimization. Maximization of entropy increases the heterogeneity of the portfolio and makes asset allocation more practicable than models without entropy. The proposed model is a multi-objective model with three objective functions that maximizes the mean return, minimizes the absolute deviation and maximizes the entropy of the portfolio, which has not been studied by past researchers. Furthermore, the proposed multi-objective model incorporates the optimal values of each objective function and is solved using a goal-programming approach. Section 2 describes the research development, methodology of the MAD model and the proposed model. Section 3 presents the empirical results of this research and discussion. The conclusion is presented in the last section of the paper.

## 2. Methodology

### 2.1. Research Development

**Figure 1:**
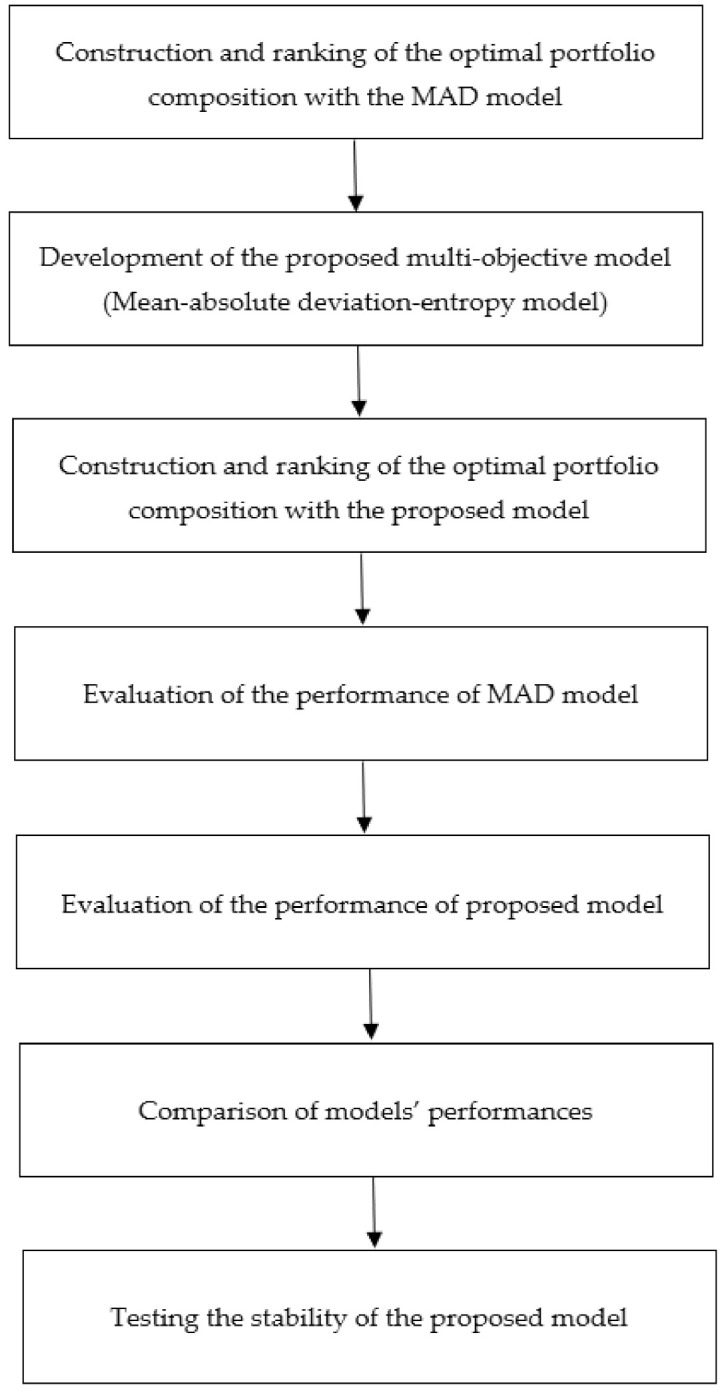
Flowchart of the research framework.

In this research, we propose a multi-objective model for portfolio optimization, namely mean-absolute deviation-entropy model. Figure 1 presents a flowchart of the research framework in this paper.

Based on Figure 1, the details of the research framework are presented as follows:

Step 1: Construction and ranking of the composition of the optimal portfolio with the MAD model. Firstly, the optimal portfolio is constructed with the MAD model. Next, the stocks are ranked based on weight, in descending order.

Step 2: Development of the proposed multi-objective model, namely a mean-absolute deviation-entropy model, by incorporating the maximization of entropy as an objective function. In addition, the proposed model incorporates the optimal value of each objective function using a goal-programming approach. The objective functions of the proposed model are to maximize the mean return, minimize the absolute deviation and maximize the entropy of the portfolio.

Step 3: Construction and ranking of the composition of the optimal portfolio with the proposed model. Firstly, the optimal portfolio is constructed with the proposed model. Next, the stocks are ranked based on weight, in descending order.

Step 4: Evaluation of the performance of the MAD model. The optimal portfolio performance of the MAD model is measured using performance ratio.

Step 5: Evaluation of the performance of the proposed model. The optimal portfolio performance of the proposed model is measured using performance ratio.

Step 6: Comparison of the models’ performances. The optimal portfolio performance among the MAD model, proposed model and benchmark naive diversification strategy, are compared using performance ratio.

Step 7: Testing the stability of the proposed model. Simulation analyses are performed on the portfolio return of the MAD model, proposed model and the naive diversification strategy using bootstrap simulation. Based on the simulation analyses, the optimal portfolio performance among the MAD model, proposed model and the naive diversification strategy are compared using performance ratio.

The MAD model, proposed model and the naive diversification strategy are tested with a numerical example that consists of weekly returns of stocks of the Dow Jones Industrial Average (DJIA), which are listed in the New York Stock Exchange (NYSE), by comparing the performance of the optimal portfolio for the two periods, which are January 2016–December 2019 (before the COVID-19 pandemic period) and January 2020–March 2021 (within the COVID-19 pandemic period). The returns of stocks of the DJIA have been used as data in studies on portfolio optimization by past researchers [27,28,29].

The methodologies of the MAD model and the proposed model are presented in Section 2.2 and Section 2.3, respectively. Table 1 presents the symbols of the MAD model and the proposed model used in this study.

### 2.2. Mean-Absolute Deviation Model

Konno and Yamazaki [10] have proposed the absolute-deviation risk function, as shown in Equation (1), to replace the standard-deviation risk function, σ(x), of Markowitz [4].

(1) $$w(x)=E[\left|\sum_{j=1}^{n}R_{j}x_{j}-E[\sum_{j=1}^{n}R_{j}x_{j}]\right|]$$

Minimizing w(x) is equivalent to minimizing σ(x) if $\left(R_{1}\ldots ,R_{n}\right)$ are multivariate and normally distributed, leading to the following MAD model:

(2) Minimize w(x),

subject to

(3) $$\sum_{j=1}^{n}E[R_{j}]x_{j}\geq \rho ,$$

(4) $$\sum_{j=1}^{n}x_{j}=1,$$

(5) $$x_{j}\geq 0_{,}j=1,\ldots ,n.$$

In the MAD model, the objective function is to minimize the absolute deviation of the portfolio.w(x) can be approximated as follows:

(6) $$w(x)=\frac{1}{T}\sum_{t=1}^{T}\left|\sum_{j=1}^{n}(r_{jt}-r_{j})x_{j}\right|$$

Denoting

(7) $$a_{jt}=r_{jt}-r_{j}$$

Assume $r_{jt}$ to be the realization of random variable $R_{j}$ during period *t* (*t* = 1,2,...,*T*).

The model (2)–(5) converts to the following model:

(8) $$\mathrm{Minimize}\;\sum_{t=1}^{T}\left|\sum_{j=1}^{n}a_{jt}x_{j}\right|/T$$

(9) $$\mathrm{subject}\;\mathrm{to}\;\sum_{j=1}^{n}r_{j}x_{j}\geq \rho ,$$

Equations (4) and (5).

The model above [(8), (9), (4), (5)] is equivalent to the linear programming model as shown below:

(10) $$\mathrm{Minimize}\;\sum_{t=1}^{T}y_{t}/T$$

(11) $$\mathrm{subject}\;\mathrm{to}\;y_{t}+\sum_{j=1}^{n}a_{jt}x_{j}\geq 0,t=1,\ldots ,T,$$

(12) $$y_{t}-\sum_{j=1}^{n}a_{jt}x_{j}\geq 0,t=1,\ldots ,T,$$

Equations (4), (5) and (9).

According to Konno and Yamazaki [10], short sale is not allowed for the MAD model as shown in Equation (5).

### 2.3. Proposed Mean-Absolute Deviation-Entropy Model

Shannon [19] has introduced entropy, H(x), as the uncertainty measure, as shown in Equation (13).

(13) $$H(x)=-\sum_{j=1}^{n}x_{j}\ln x_{j},$$

The main advantage of entropy is to generate a well-diversified portfolio. As shown in Equation (13), the entropy function is a concave function of the portfolio weights $x_{1},\ldots x_{n}$, which has its maximum value ln*n*, when $x_{j}=\frac{1}{n}$ for j=1,…,n. Therefore, due to this property, entropy is a good indicator of diversity in a probability distribution for generation of a well-diversified portfolio.

Another advantage of the principle of maximum entropy is the prevention of extreme weight on asset allocation due to a very small number of assets in portfolio selection. For the extreme case, according to Equation (13), the entropy function achieves the minimum value 0, when $x_{j}=1$ and $x_{i}=0$, i≠j for i=1,…,n. This implies that the entropy achieves the minimum value 0 when only one asset is selected in the portfolio. Therefore, a higher value of the entropy measure for portfolio weights will give higher portfolio diversification. Diversification can reduce unsystematic risk in the portfolio.

Additionally, the incorporation of entropy as an objective function in the optimization model can increase the diversification degree of the optimal portfolio. A multi-objective optimization model, namely mean-absolute deviation-entropy model is proposed in this section by incorporating the maximization of entropy. This is because the incorporation of entropy can diversify the allocation of various assets in portfolio selection. The proposed multi-objective optimization model consists of three objective functions that focus on the mean return, absolute deviation and entropy of the portfolio. The three objective functions of the proposed model are to maximize the mean return, minimize the absolute deviation and maximize the entropy of the portfolio. In addition, the proposed multi-objective optimization model incorporates the optimal value of each objective function and is solved using a goal-programming approach. This is because the goal-programming approach can solve multi-objective problems [30,31,32]. The proposed model is formulated as follows:

(14) $$\mathrm{Maximize}\;R(x)=\sum_{j=1}^{n}E[R_{j}]x_{j},$$

(15) Minimize w(x),

(16) Maximize H(x),

subject to

(17) $$\sum_{j=1}^{n}x_{j}=1,$$

(18) $$x_{j}\geq 0_{,}j=1,\ldots ,n.$$

The optimal value for portfolio mean return, R*, can be obtained by solving the following model.

(19) Maximize R(x),

$$\begin{array}{c} \mathrm{subject}\;\mathrm{to}\\ \mathrm{Equations}\;(17)\;\mathrm{and}\;(18). \end{array}$$

The optimal value for portfolio absolute deviation, W*, can be determined by solving the following model.

(20) Minimizew(x),

$$\begin{array}{c} \mathrm{subject}\;\mathrm{to}\\ \mathrm{Equations}\;(17)\;\mathrm{and}\;(18). \end{array}$$

Then, the optimal values R* and W* are substituted into the following model based on the goal-programming approach:

(21) $$\mathrm{Minimize}\;Z=\left|\frac{d_{1}}{R*}\right|+\left|\frac{d_{2}}{W*}\right|+\left|\frac{d_{3}}{\ln n}\right|,$$

subject to

(22) $$R(x)+d_{1}=R*,$$

(23) $$w(x)-d_{2}=W*,$$

(24) $$H(x)+d_{3}=\ln\;n,$$

Equations (17) and (18).

The target value for portfolio entropy is set as ln*n,* because it is the maximum value of portfolio entropy. The objective function of the proposed model is to minimize deviations from the optimal values R*, W* and target value, ln*n* using a goal-programming approach.

Short sale is not allowed for the proposed model as shown in Equation (18). This is because the proposed model is developed based on the MAD model [10] and the entropy that has been introduced by Shannon [19]. Both the MAD model and entropy function require positive weights of assets as shown in Equations (5) and (13), respectively. These are the limitations of the proposed model.

### 2.4. Model Performance

The performance of the model is measured with performance ratio. The performance ratio is measured based on the mean-absolute deviation ratio [10] as shown in Equation (25). Higher performance ratio indicates higher performance of the model.

(25) $$\mathrm{Performance}\;\mathrm{ratio}\;=\frac{\mathrm{Portfolio}\;\mathrm{mean}\;\mathrm{return}}{\mathrm{Portfolio}\;\mathrm{absolute}\;\mathrm{deviation}}$$

The computational works of the MAD model and the proposed model are performed using LINGO software. The performance of the optimal portfolio for the MAD model, the proposed model and benchmark naive diversification strategy (1/*N* portfolio) are compared for the two periods which are January 2016–December 2019 (before the COVID-19 pandemic period) and January 2020–March 2021 (within the COVID-19 pandemic period). The January 2016–December 2019 period (before the COVID-19 pandemic period) is denoted by D1 period, while the January 2020–March 2021 period (within the COVID-19 pandemic period) is denoted by D2 period.

### 2.5. Bootstrap Simulation

In this study, we test the stability of the proposed model using bootstrap simulation [33,34]. Bootstrap simulation is an important financial tool in portfolio optimization [34,35]. We simulate the portfolio return of the proposed model, the MAD model and the naive diversification strategy (1/*N* portfolio). Bootstrapping is repeated 5000 times for the tested D1 period and D2 period in this study with *R* software. Based on the simulation analyses, the performance of the optimal portfolios among the MAD model, the proposed model and the naive diversification strategy are compared using performance ratio.

## 3. Results and Discussion

### 3.1. Descriptive Statistics of the Returns of Stocks

Table 2 displays descriptive statistics of the returns of stocks of the DJIA that are used as the data of this study.

As shown in Table 2, the descriptive statistics of returns of stocks which are mean, standard deviation, skewness and kurtosis, are presented. AAPL gives the highest mean (0.0063) while WBA gives the lowest mean (−0.0010). In addition, BA has the highest standard deviation (0.0775), whereas PG has the lowest standard deviation (0.0249). Furthermore, BA exhibits the highest skewness (2.1565), while KO exhibits the lowest skewness (−1.5923). Moreover, BA shows the highest kurtosis (31.7449), whereas AMGN shows the lowest kurtosis (0.5722). This implies that the mean, standard deviation, skewness and kurtosis of the returns of stocks are different.

### 3.2. Optimal Portfolio Composition

Table 3 presents the composition and ranking of stocks in the optimal portfolio that was constructed with the MAD model for the D1 period and D2 period.

As presented in Table 3, PG is ranked the highest because it gives the largest weight to the portfolio composition using the MAD model for the D1 period. Conversely, VZ is ranked the lowest as it gives the smallest weight to the portfolio composition using the MAD model for the D1 period.

For the D2 period, VZ is ranked the highest since it gives the largest weight to the portfolio composition using the MAD model. In contrast, JNJ is ranked the lowest as it gives the smallest weight to the portfolio composition using the MAD model for the D2 period.

Table 4 presents the composition and ranking of stocks in the optimal portfolio that was constructed with the proposed mean-absolute deviation-entropy model for the D1 period and D2 period.

Based on Table 4, MSFT is ranked the highest because it gives the largest weight to the portfolio composition using the proposed model for the D1 period. In contrast, WBA is ranked the lowest as it gives the smallest weight to the portfolio composition using the proposed model for the D1 period.

According to Table 4, MSFT is ranked the highest because it gives the largest weight to the portfolio composition using the proposed model for the D2 period. Conversely, BA is ranked the lowest as it gives the smallest weight to the portfolio composition using the proposed model for the D2 period.

The MAD model (Table 3) and the proposed model (Table 4) led to different compositions of the optimal portfolio in both the D1 period and the D2 period. This implies that the incorporation of entropy in the proposed model will change the optimal portfolio composition. It shows that the proposed model is able to generate a well-diversified portfolio. This is because the proposed model gives a higher portfolio entropy value than the MAD model for the D1 period and the D2 period. Based on Equation (13), the portfolio entropy values of the MAD model and the proposed model are 2.2156 and 2.7054, respectively, for the D1 period. Furthermore, the portfolio entropy values of the MAD model and the proposed model are 1.6357 and 2.5124, respectively, for the D2 period.

Table 3 indicates that the ranking of all stocks in the optimal portfolio of the MAD model for the D1 period and D2 period are different. The symbol—implies that the stock is not selected in the optimal portfolio of the MAD model for the D1 period and D2 period. AAPL, CAT and JNJ are not selected in the optimal portfolio of the MAD model for the D1 period. On the other hand, AXP, BA, CVX, KO, JPM, MRK, PG, CRM and UNH are not selected in the optimal portfolio of the MAD model for the D2 period. Furthermore, Table 4 indicates that the ranking of all stocks in the optimal portfolio of the proposed model for the D1 period and D2 period are different, except for MSFT.

### 3.3. Model Performance

Table 5 shows the performance comparison of the optimal portfolios among the MAD model, the proposed model and the naive diversification strategy for the D1 period.

Based on Table 5, the MAD model generates the portfolio mean return at 0.3000% for the D1 period. Furthermore, the portfolio performance ratio of the MAD model is 0.2970. The performance ratio is measured based on the mean-absolute deviation ratio.

On the other hand, the proposed model generates the portfolio mean return at 0.4000% for the D1 period. In addition, the portfolio performance ratio of the proposed model is 0.3254.

Besides that, the naive diversification strategy generates the portfolio mean return at 0.2425% for the D1 period. Moreover, the portfolio performance ratio of the naive diversification strategy is 0.2245.

Table 6 shows the performance comparison of the optimal portfolios among the MAD model, the proposed model and the naive diversification strategy for the D2 period.

Based on Table 6, the MAD model generates the portfolio mean return at 0.3400% for the D2 period. Furthermore, the portfolio performance ratio of the MAD model is 0.1762. The performance ratio is measured based on the mean-absolute deviation ratio.

On the other hand, the proposed model generates the portfolio mean return at 0.4400% for the D2 period. In addition, the portfolio performance ratio of the proposed model is 0.2041.

Finally, the naive diversification strategy generates the portfolio mean return at 0.3185% for the D2 period. Moreover, the portfolio performance ratio of the naive diversification strategy is 0.1313.

The proposed model outperforms the MAD model and the naive diversification strategy by giving a higher performance ratio for the D1 period (Table 5) and the D2 period (Table 6). In addition, the proposed model generates higher portfolio mean return than the MAD model and the naive diversification strategy for both periods. This is because the proposed model is a multi-objective model that maximizes the mean return, minimizes the absolute deviation and maximizes the entropy of the portfolio. Moreover, the proposed model incorporates the optimal value of each objective function and is solved using a goal-programming approach.

### 3.4. Simulation Analyses

Table 7 presents the bootstrap simulation analysis on the portfolio return of the MAD model, proposed model and the naive diversification strategy for the D1 period.

As shown in Table 7, the MAD model generates the portfolio mean return at 0.2984% with a 95% confidence interval of 0.2955–0.3012% for the D1 period. The portfolio performance ratio of the MAD model is 0.2909. The proposed model generates the portfolio mean return at 0.4011% with a 95% confidence interval of 0.3979–0.4043% for the D1 period. The portfolio performance ratio of the proposed model is 0.3798. On the other hand, the naive diversification strategy generates the portfolio mean return at 0.2445% with a 95% confidence interval of 0.2416–0.2474% for the D1 period. The portfolio performance ratio of the naive diversification strategy is 0.2340.

The simulation analysis shows that the proposed model outperforms the MAD model and the naive diversification strategy by giving a higher performance ratio for the D1 period. In addition, the proposed model generates a higher portfolio mean return than the MAD model and the naive diversification strategy for the D1 period. The simulation analysis is consistent with the results for actual portfolio mean return and performance ratio, which are presented in Table 5.

Table 8 presents the bootstrap simulation analysis on the portfolio return of the MAD model, proposed model and the naive diversification strategy for the D2 period.

Based on Table 8, the MAD model generates the portfolio mean return at 0.3507% with a 95% confidence interval of 0.3417–0.3597% for the D2 period. The portfolio performance ratio of the MAD model is 0.1927. The proposed model generates the portfolio mean return at 0.4341% with a 95% confidence interval of 0.4234–0.4447% for the D2 period. The portfolio performance ratio of the proposed model is 0.2242. In contrast, the naive diversification strategy generates the portfolio mean return at 0.3073% with a 95% confidence interval of 0.2947–0.3199% for the D2 period. The portfolio performance ratio of the naive diversification strategy is 0.1635.

Based on the simulation analysis, the proposed model outperforms the MAD model and the naive diversification strategy by giving a higher performance ratio for the D2 period. Furthermore, the proposed model generates a higher portfolio mean return than the MAD model and the naive diversification strategy for the D2 period. This shows that the simulation analysis is consistent with the results for actual portfolio mean return and performance ratio, which are presented in Table 6.

The simulation analyses show that proposed model generates a higher portfolio mean return and a higher performance ratio than the MAD model, as well as the naive diversification strategy, consistently for both periods of study, which are before the COVID-19 pandemic period (Table 7) and within the COVID-19 pandemic period (Table 8). This implies that the proposed model is robust and stable for portfolio investment. This is because the proposed model is a multi-objective model that maximizes the mean return, minimizes the absolute deviation and maximizes the entropy of the portfolio. Furthermore, the proposed model incorporates the optimal value of each objective function and is solved using a goal-programming approach.

## 4. Conclusions

In conclusion, a multi-objective optimization model, namely a mean-absolute deviation-entropy model is proposed in this paper by incorporating the entropy as well as the optimal value of each objective function using a goal-programming approach. The results indicate that the MAD model and the proposed model give different portfolio composition for both periods of study, which are before the COVID-19 pandemic period as well as within the COVID-19 pandemic period. In addition, the results show that the proposed model is able to generate well-diversified portfolios consistently, because the proposed model gives a higher portfolio entropy value than the MAD model for both periods of study. The incorporation of entropy in the proposed model will change the optimal portfolio composition in order to generate well-diversified portfolio.

The proposed model outperforms the MAD model and the naive diversification strategy by giving higher performance ratios consistently for both periods of study. Besides that, the proposed model generates higher portfolio mean returns than the MAD model and the naive diversification strategy consistently, both before the COVID-19 pandemic period as well as within the COVID-19 pandemic period. Based on the simulation analyses, the proposed model generates higher portfolio mean returns and higher performance ratios than the MAD model, as well as the naive diversification strategy, consistently for both periods of study. This implies that the proposed model is robust and stable for portfolio investment.

As presented in this paper, the proposed mean-absolute deviation-entropy model is superior to both the MAD model and the naive diversification strategy, as it gives higher performance ratios and higher portfolio mean returns. This study will contribute to the portfolio management of investors. This is because investors will be able to maximize the portfolio mean return, minimize the portfolio absolute deviation and maximize the portfolio entropy, therefore generating well-diversified portfolios and reducing the unsystematic risk of the portfolios using the proposed model. Portfolio optimization with the proposed model should be extended to the stock market of the other countries for future research. This proposed model can be applied using data from other markets, be it developed, emerging, or developing markets, with promising results, benefiting investors in various countries.

## Figures and Tables

**Table 1:** Symbols of the MAD model and the proposed model.

Symbol	Description
$R_{j}$	return of asset *j*
$x_{j}$	weight of asset *j*
ρ	minimum return set by the investor
$R\left(x\right)$	portfolio mean return
$w\left(x\right)$	portfolio absolute deviation
$H\left(x\right)$	portfolio entropy
$R^{*}$	optimal value for portfolio mean return
$W^{*}$	optimal value for portfolio absolute deviation
$d_{1}$	deviation of portfolio mean return from the optimal value
$d_{2}$	deviation of portfolio absolute deviation from the optimal value
$d_{3}$	deviation of portfolio entropy from the optimal value

**Table 2:** Descriptive statistics of returns of stocks.

Stocks	Mean	Standard Deviation	Skewness	Kurtosis
MMM	0.0015	0.0322	−0.7917	2.1866
AXP	0.0037	0.0474	0.1840	10.4748
AMGN	0.0022	0.0349	−0.0553	0.5722
AAPL	0.0063	0.0393	−0.2796	2.8098
BA	0.0048	0.0775	2.1565	31.7449
CAT	0.0053	0.0418	−0.2800	1.4749
CVX	0.0014	0.0405	−1.1459	11.2163
CSCO	0.0030	0.0338	−0.2415	1.9929
KO	0.0012	0.0293	−1.5923	12.5853
GS	0.0031	0.0449	0.4427	5.7500
HD	0.0038	0.0381	−0.3786	14.4991
HON	0.0035	0.0353	−0.8228	11.6518
IBM	0.0006	0.0350	−0.2287	3.6550
INTC	0.0031	0.0411	−0.3729	2.6244
JNJ	0.0020	0.0257	−0.4792	2.5670
JPM	0.0039	0.0396	0.1057	6.3517
MCD	0.0028	0.0290	−0.7580	7.1262
MRK	0.0018	0.0284	−0.0812	0.9399
MSFT	0.0057	0.0301	−0.4318	2.5895
NKE	0.0034	0.0380	0.6587	5.3541
PG	0.0022	0.0249	−0.1018	3.4584
CRM	0.0046	0.0447	1.1580	8.4621
TRV	0.0017	0.0354	0.1498	6.1128
UNH	0.0050	0.0400	−0.5416	7.4587
VZ	0.0011	0.0254	0.1536	1.0952
V	0.0042	0.0310	−0.6160	5.6441
WBA	−0.0010	0.0408	−0.0805	2.0924
WMT	0.0033	0.0280	0.1636	2.5789
DIS	0.0027	0.0354	0.0463	4.4153

**Table 3:** Composition and ranking of stocks in the optimal portfolio (MAD model) for the D1 period and D2 period.

Stocks	Weights (%) [D1 Period]	Ranking [D1 Period]	Weights (%) [D2 Period]	Ranking [D2 Period]
AXP	0.7904	12	-	-
AAPL	-	-	2.9198	6
BA	2.3983	10	-	-
CAT	-	-	10.1117	4
CVX	11.2762	4	-	-
KO	9.5720	5	-	-
JNJ	-	-	1.4497	7
JPM	6.0708	7	-	-
MCD	15.8696	3	7.6486	5
MRK	4.5878	8	-	-
MSFT	3.7366	9	23.4267	2
PG	18.5039	1	-	-
CRM	1.2556	11	-	-
UNH	9.2145	6	-	-
VZ	0.0759	13	31.1050	1
WMT	16.6482	2	23.3385	3

**Table 4:** Composition and ranking of stocks in the optimal portfolio (Proposed model) for the D1 period and D2 period.

Stocks	Weights (%) [D1 Period]	Ranking [D1 Period]	Weights (%) [D2 Period]	Ranking [D2 Period]
MMM	0.0287	27	4.0459	9
AXP	0.9634	20	0.0246	26
AMGN	0.2127	25	1.4396	15
AAPL	7.7781	6	4.0520	8
BA	7.2796	7	0.77584 × 10 ^−7^	29
CAT	1.3159	14	8.6001	4
CVX	1.0525	17	0.0140	28
CSCO	0.3132	22	1.8066	14
KO	1.3468	13	0.1865	22
GS	0.0261	28	0.8782	17
HD	1.2611	15	3.0300	10
HON	0.9761	19	0.0896	24
IBM	0.0316	26	0.4680	19
INTC	0.3000	23	0.4319	20
JNJ	1.0515	18	4.3079	7
JPM	3.1691	11	0.6681	18
MCD	6.6089	8	4.9987	5
MRK	4.3922	9	1.3984	16
MSFT	15.1745	1	25.6371	1
NKE	1.1007	16	2.3731	12
PG	8.1654	5	1.9720	13
CRM	3.8759	10	2.4811	11
TRV	0.2590	24	0.0879	25
UNH	10.6100	3	0.2193	21
VZ	1.7637	12	13.2411	2
V	9.5210	4	0.1586	23
WBA	0.0015	29	0.0175	27
WMT	11.0766	2	12.5844	3
DIS	0.3443	21	4.7879	6

**Table 5:** Performance comparison of the optimal portfolios among the MAD model, the proposed model and the naive diversification strategy for the D1 period.

Optimal Portfolio	MAD Model	Proposed Model	Naive Diversification Strategy
Portfolio mean return (%)	0.3000	0.4000	0.2425
Performance ratio	0.2970	0.3254	0.2245

**Table 6:** Performance comparison of the optimal portfolios among the MAD model, the proposed model and the naive diversification strategy for the D2 period.

Optimal Portfolio	MAD Model	Proposed Model	Naive Diversification Strategy
Portfolio mean return (%)	0.3400	0.4400	0.3185
Performance ratio	0.1762	0.2041	0.1313

**Table 7:** Bootstrap simulation analysis on the portfolio return of the MAD model, proposed model and the naive diversification strategy for the D1 period.

Optimal Portfolio	MAD Model	Proposed Model	Naive Diversification Strategy
Portfolio mean return (%)	0.2984	0.4011	0.2445
95% Confidence Interval	0.2955–0.3012	0.3979–0.4043	0.2416–0.2474
Performance ratio	0.2909	0.3798	0.2340

**Table 8:** Bootstrap simulation analysis on the portfolio return of the MAD model, proposed model and the naive diversification strategy for the D2 period.

Optimal Portfolio	MAD Model	Proposed Model	Naive Diversification Strategy
Portfolio mean return (%)	0.3507	0.4341	0.3073
95% Confidence Interval	0.3417–0.3597	0.4234–0.4447	0.2947–0.3199
Performance ratio	0.1927	0.2242	0.1635
